# Mesothelin's minimal MUC16 binding moiety converts TR3 into a potent cancer therapeutic *via* hierarchical binding events at the plasma membrane

**DOI:** 10.18632/oncotarget.8925

**Published:** 2016-04-22

**Authors:** Yang Su, Katharina Tatzel, Xuejun Wang, Brian Belt, Pratibha Binder, Lindsay Kuroki, Matthew A. Powell, David G. Mutch, William G. Hawkins, Dirk Spitzer

**Affiliations:** ^1^ Department of Surgery, Washington University School of Medicine, St. Louis, Missouri 63110, USA; ^2^ Department of General Surgery, Shengjing Hospital of China Medical University, Shenyang, Liaoning 110004, China; ^3^ Division of Gynecologic Oncology, Washington University School of Medicine, St. Louis, Missouri 63110, USA; ^4^ Alvin J. Siteman Cancer Center, St. Louis, Missouri 63110, USA

**Keywords:** TRAIL, Meso64-TR3, mesothelin, MUC16, CA125

## Abstract

TRAIL has been extensively explored as a cancer drug based on its tumor-selective activity profile but it is incapable *per se* of discriminating between death receptors expressed by normal host cells and transformed cancer cells. Furthermore, it is well documented that surface tethering substantially increases its biologic activity. We have previously reported on Meso-TR3, a constitutive TRAIL trimer targeted to the biomarker MUC16 (CA125), in which the entire ectodomain of human mesothelin was genetically fused to the TR3 platform, facilitating attachment to the cancer cells via the MUC16 receptor. Here, we designed a truncation variant, in which the minimal 64 amino acid MUC16 binding domain of mesothelin was incorporated into TR3. It turned out that the dual-domain biologic Meso64-TR3 retained its high MUC16 affinity and bound to the cancer cells quickly, independent of the TR3/death receptor interaction. Furthermore, it was substantially more potent than Meso-TR3 and TR3 *in vitro* and in a preclinical xenograft model of MUC16-dependent ovarian cancer. Phenotypically, Meso64-TR3 is more closely related to non-targeted TR3, evident by indistinguishable activity profiles on MUC16-deficient cancers and similar thermal stability characteristics. Overall, Meso64-TR3 represents a fully human, MUC16-targetd TRAIL-based biologic, ideally suited for exploring preclinical and clinical evaluation studies in MUC16-dependent malignancies.

## INTRODUCTION

Tumor necrosis factor-related apoptosis-inducing ligand (TRAIL) was discovered in the mid 1990's as a new member of the large tumor necrosis factor (TNF) superfamily and caught immediate attention as a promising cancer therapeutic [1–4]. Viewed as the most favorable property of TRAIL as a drug candidate was the fact that it selectively induced apoptosis in transformed tumor cells but not in normal cells *in vivo*, without causing toxicity following systemic applications [5–7], one of the key discoveries among members of the TNF superfamily [8, 9]. TRAIL exerts its biological functions via binding to cell surface-expressed death receptors DR4 and DR5 [10–14], which triggers cell death through activation of the extrinsic apoptosis pathway [15], mediated via death receptor clustering and the formation of the death-inducing signaling complex (DISC) with the involvement of the initiator caspase-8 and the executioner caspase-3, ultimately leading to programmed cell death [16–19]. Moreover, it turned out that TRAIL acted independently of p53, which suggested that chemotherapy-resistant tumors caused by inactivating mutations of this tumor suppressor were still sensitive to TRAIL-based therapies [11, 20, 21]. Based on these features, a number of clinical trials have been initiated, while numerous attempts to develop more potent TRAIL variants were concurrently explored, including stabilization with trimerization domains (leucine zipper [LZ]), formation of higher-order TRAIL complexes and genetic fusions with the constant regions (Fc) of human immunoglobulins [22–24].

In this regard, we have pioneered an entirely new concept to generate constitutively trimerized TRAIL biologics via genetic engineering. This novel drug design was created by covalently linking three TRAIL ectodomains into a single fusion protein, designated TR3, characterized by enhanced stability and apoptosis induction capacity and the ability for downstream modification options in a modular and stoichiometrically fully-controlled fashion [25]. The latter aspect has far reaching consequences with respect to developing truly tumor-targeted TR3 biologics, selective for a given cell surface biomarker. This targeting concept is particularly attractive not only for the site-specific delivery and the accumulation of the therapeutics at the tumor cell membrane, it converts soluble TR3 drugs into membrane-bound analogs, a process which substantially increases death receptor signaling and thereby the overall bioactivity of the therapeutic [26]. More specifically, by taking advantage of the high affinity interaction between mesothelin and MUC16 [27], we recently designed a mesothelin/TR3 fusion protein, designated Meso-TR3, in order to tether our therapeutic to the MUC16 biomarker located on the tumor cell membrane [28].

Even though Meso-TR3 demonstrated several favorable properties, such as improved bioactivity on MUC16-expressing tumors *in vitro* and *in vivo*, we suspected that it's relatively large molecular weight could prove prohibitive when it comes to drug penetration into solid tumors, as these are often characterized by extensive stromal components, especially relevant in pancreatic cancer [29, 30]. Based on this consideration and the notion that the amino-terminal 64 amino acids of mesothelin have been described to be sufficient to facilitate binding to native MUC16 [Ref. [31]], we designed a Meso-TR3 truncation variant, designated Meso64-TR3. Here, we describe the properties of this re-designed, MUC16-targeted TR3 trimer in a head-to-head comparison with its full length first-generation predecessor and demonstrate its unaltered MUC16 binding capacity combined with improved stability and superior biologic activity. We identified the high affinity of the mesothelin/MUC16 interaction as the dominant parameter for the rapid attachment of targeted TR3 fusion proteins to MUC16-positive cancer cells with the TR3/DR interaction playing a secondary role. We thus believe that Meso64-TR3 will be widely applicable for the treatment of MUC16-positive malignancies, including ovarian, breast and pancreatic cancers [32–34].

## RESULTS

### Meso64-TR3 retains strong binding capacity to MUC16-expressing cancer cells

**Figure 1:**
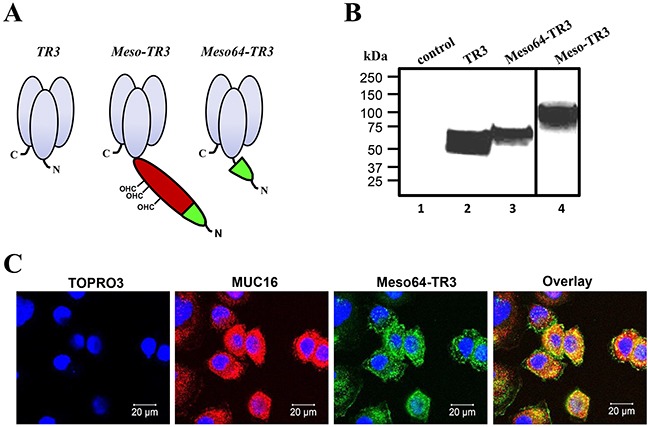
Design and binding profile of targeted Meso64-TR3 on MUC16-expressing cancer cells **A.** Schematic representation of the proteins designed for this study. All proteins are based on the TR3 drug platform. The first generation, MUC16-targeted TRAIL trimer Meso-TR3 contains the entire mesothelin ectodomain as delivery vehicle (red and green; CHO, O-linked glycosylation sites). Meso64-TR3 represents a MUC16-targeted TR3 trimer in which only the 64 amino-terminal amino acids of mesothelin were used as delivery moiety (green). Both targeted biologics contain N-terminal FLAG tags for immunologic detection purposes. **B.** Western blot analysis (reducing conditions) documents the molecular weights of TR3 (≈60 kDa, lane 2), Meso-TR3 (≈100 kDa, lane 4) and Meso64-TR3 (≈65 kDa, lane 3) using anti-TRAIL pAb. Supernatant from mock-transfected HEK293T cells served as a negative control (lane 1). **C.** MUC16-expressing OVCAR3 cells were grown on 8-chamber EZ slides and incubated the following day with Meso64-TR3 complexed with DR5-Fc. After washing, the cells were stained with anti-MUC16 pAb (red) and anti-FLAG mAb (green), respectively. The cells were counterstained with TOPRO3 (blue, nuclei) and analyzed by confocal microscopy. The individual channels were overlaid to document co-localization of the tumor marker and the targeted cancer drug (Overlay). Original magnification: 40x.

Our previously described MUC16-targeted cancer drug Meso-TR3 contained the peptide sequence of the entire mature ectodomain of human mesothelin fused to the N-terminus of TR3 [28]. However, Meso-TR3 is a rather bulky molecule and contains several mesothelin-derived glycosylation sites, which heavily contribute to its large molecular weight. Along these lines, and in an effort to reduce the molecular weight of MUC16-targeted Meso-TR3, it has been shown that the 64 N-terminal amino acids of mesothelin are sufficient to facilitate strong interaction with MUC16 [35]. These considerations prompted us to design a truncation variant by inserting the corresponding 64 amino acid mesothelin-encoding cDNA into the 5′-terminus of the TR3 expression platform (Figure 1A). Both MUC16-targeted recombinant fusion proteins contain an N-terminal FLAG epitope tag for immunologic detection purposes (not shown). The proteins were produced in HEK293T cells and their molecular weight was confirmed by Western blot analysis. With ≈65 kDa, Meso64-TR3 was only ≈5 kDa larger (+8%) than parental TR3 (≈61 kDa) and ≈35 kDa smaller (−35%) than Meso-TR3 (≈100 kDa) (Figure 1B).

Due to the drastically reduced targeting domain, an initial concern was the ability of Meso64-TR3 to interact efficiently with native MUC16. We therefore performed confocal microscopy following drug exposure employing the MUC16-positive ovarian cancer cell line OVCAR3. Since Meso64-TR3 represents a dual-domain therapeutic (a MUC16 interacting-, and a death receptor effector domain), two possible binding mechanisms had to be distinguished: the TR3/death receptor interaction (DR4, DR5, DcR1 and DcR2) and the mesothelin/MUC16 interaction. In order to prevent drug binding via the TR3/DR interaction, Meso64-TR3 was complexed with soluble death receptor 5 (DR5-Fc), prior to exposure to the cancer cells [28]. Confocal microscopy confirmed a signal overlap between the MUC16 marker and surface-tethered Meso64-TR3 (Figure 1C). These initial results were highly encouraging and suggested that the minimal MUC16 binding domain of human mesothelin (amino acids 1 - 64) was indeed sufficient to tether Meso64-TR3 to the OVCAR3 cell membrane. Similar binding results were obtained in other cancer cell types, such as HeLa (cervical cancer) and HPAC (pancreatic cancer), both characterized by more heterogenous MUC16 expression profiles (see below).

### Meso64-TR3 shares functional similarity with TR3 on MUC16-negative cells but is a much stronger apoptosis-inducer than Meso-TR3 on MUC16-expressing cancer cells

**Figure 2:**
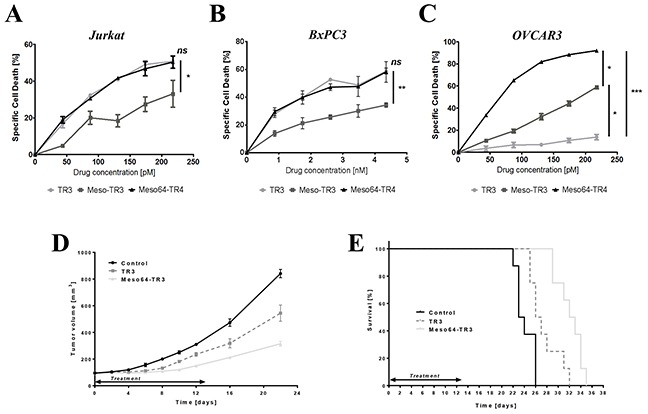
Meso64-TR3 unleashes its potency on MUC16-expressing tumor cells *in vitro* and *in vivo* **A.** Cell killing profiles of TR3, Meso-TR3 and Meso64-TR3 at equimolar concentration ranges were established on the MUC16-deficient T cell leukemia cell line Jurkat. NS, not significant; *, *P* < 0.03. **B.** Similar result was repeated on another nearly MUC16-deficient pancreatic cancer cell line BxPC3. NS, not significant; **, *P* < 0.007. **C.** The same killing assay as in (A) using identical drug concentrations but the MUC16-positive ovarian cancer cell line OVCAR3 instead. *, *P* < 0.02; ***, *P* < 0.0002. **D.** Nude mice with established subcutaneous flank tumors were treated daily for 13 days with 655 pmoles TR3, Meso64-TR3 and PBS only (control). Tumor sizes were measured using electronic calipers. ****, *P* < 0.0001. **E.** Kaplan-Mayer survival curve of the drug-treated mice shown in (D). Mice were considered dead after the tumors exceeded 1000 mm^3^. ****, *P* < 0.0001.

Our TRAIL-based biologics are characterized by a multi-domain architecture, a biomarker recognition domain (mesothelin) and the activation domain of the extrinsic death pathway (TR3). In an attempt to shed light on the isolated functionality of the TR3 effector domain, we treated TRAIL-sensitive, MUC16-deficient Jurkat cells with our biologics. All of our drugs induced target cell death in a dose-dependent fashion. Of note, at equimolar concentrations, Meso64-TR3's activity profile exactly matched that of non-targeted TR3, whereas Meso-TR3's potency was substantially reduced, consistent with our previous report (Figure 2A). Similar activity profiles were obtained for all three drugs on other cell types known to be largely devoid of MUC16-expression, again highlighting the similarity between Meso64-TR3 and non-targeted TR3 relative to Meso-TR3 (Figure 2B, BxPC3 pancreatic cancer).

Most importantly, however, when all three drugs were tested on MUC16-positive ovarian cancer cells, Meso64-TR3 was capable of eradicating nearly all target cells (92%), followed by Meso-TR3 (59%) and TR3 (14%) (Figure 2C). Moreover, when TR3 and Meso64-TR3 were tested in a preclinical model of MUC16-positive ovarian cancer, the targeted drug variant outperformed its non-targeted analog with regard to a delay in tumor growth (Figure 2D), which corresponded with a significant life extension of the animals, with median survivals of 23.5 days (control), 26.5 days (TR3) and 32.5 days (Meso64-TR3), respectively (Figure 2E). These results were very encouraging and suggest that the N-terminal 64 amino acids of mesothelin are not only sufficient to facilitate efficient binding to native MUC16, it converts Meso64-TR3 into a much more powerful cancer drug that retains its enhanced *in vitro* activity profile in a preclinical mouse model of ovarian cancer.

### Meso64-TR3-mediated cancer cell death is consistent with apoptosis

**Figure 3:**
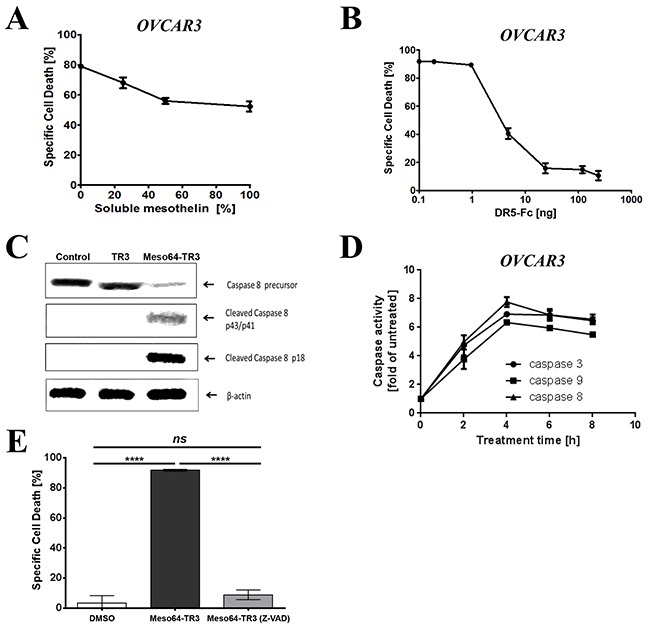
Phenotypic characterization of MUC16-targeted Meso64-TR3 **A.** OVCAR3 cells were challenged with a constant amount of Meso64-TR3 (80% specific cell death) and increasing concentrations of soluble mesothelin to study the impact of the mesothelin/MUC16 interaction of Meso64-TR3. **B.** OVCAR3 cells were challenged with a constant amount of Meso64-TR3 (90% specific cell death) and increasing concentrations of DR5-Fc to verify involvement of the extrinsic death pathway as a mechanism of Meso64-TR3-induced cell death. **C.** OVCAR3 cells were seeded in 6-well plates and treated for 4 hours with TR3, Meso64-TR3 and medium as control. The cell pellets were submitted to Western blot analysis to examine the expression and activation status of caspase-8. **D.** OVCAR3 cells were seeded in 96-well plates and treated with Meso64-TR3 for 2, 4, 6, 8 h and the activity of caspase-3, caspase-8 and caspase-9 were detected using Caspase-Glo reagent. **E.** OVCAR3 cells were treated with a constant amount of Meso64-TR3 (90% specific cell death) in the presence of Z-VAD-FMK, a pan-caspase inhibitor to block the extrinsic death pathway. Cells treated with DMSO were used as a control. Error bars, mean ± SD. Results are representatives of at least 2 independent experiments done in triplicates. NS, not significant; ****P < 0.0001.

Whenever modifications are introduced into an established drug candidate, such as TR3, it is crucial to perform a series of validation experiments to ensure that key characteristics are retained in the drug variant. These considerations also apply to the MUC16-targeted truncation variant Meso64-TR3. In order to verify that the enhanced activity profile of Meso64-TR3 was indeed related to its membrane tethering to MUC16, soluble mesothelin was used to block this interaction. In the presence of increasing concentrations of soluble mesothelin, we noticed a dose-dependent reduction in its ability to induce cell death from nearly 80% to below 53% (Figure 3A). It was further anticipated that, once attached to the cancer cell membrane, apoptosis was mediated by engagement of the TR3-effector domain with membrane-expressed death receptors, especially DR4 and/or DR5. We thus performed blocking experiments employing soluble death receptor 5 (DR5-Fc). When OVCAR3 cells were treated with Meso64-TR3 in the presence of increasing concentrations of DR5-Fc, a dose-dependent reduction of cell death was accomplished from 92% (no inhibitor) to 11% at the highest concentration of the inhibitor (Figure 3B). Similar results were seen with MUC16-deficient Jurkat cells (data not shown). These data support the notion that Meso64-TR3 does indeed require engagement with activating death receptors at the plasma cell membrane to induce cancer cell death.

The previous experiments provided circumstantial evidence for the activation of the extrinsic death pathway being responsible for the improved properties of Meso64-TR3 on MUC16-positive cancer cells. To further solidify these presumptions, we performed additional biochemical analyses regarding the key players involved in the activation and execution of apoptosis, caspases-8, -9, and -3. First, we treated OVCAR3 cells with TR3 and Meso64-TR3 for 24 h, prepared cell lysates and assessed the activation status of the most proximal signaling molecule relative to the death receptors, caspase-8, by Western blot analysis. Consistent with the strong activity profile of Meso64-TR3 on these cells, we did notice a robust induction of activated cleavage fragments of caspase-8, along with a reduction in the signal intensity of its precursor. This activation pattern was absent for both the TR3 and non-treated control cells (Figure 3C). To verify these initial results, we determined the activation profiles of caspases-8, -3 and -9 using a different assay system (Caspase-Glo assay, see M&M for details). This more quantitative analysis tool also enabled us to determine the kinetics of caspase activation. It turned out that all three caspases were activated with the same kinetics, with an activation peak around four hours post-treatment (Figure 3D). The importance of caspase activation as a mediator of Meso64-TR3-dependent cancer cell death was finally confirmed using the pan-caspase inhibitor Z-VAD-FMK. This irreversible inhibitor of intracellular caspase activation completely protected OVCAR3 cells from apoptosis (Figure 3E). Taken together, the strong death-inducing properties of Meso64-TR3 were found to depend on membrane tethering to the cancer biomarker MUC16 and was confirmed to be consistent with key attributes of death receptor-mediated, caspase-dependent forms of programmed cell death - apoptosis.

### Meso64-TR3 is a temperature-stabilized monomer

**Figure 4:**
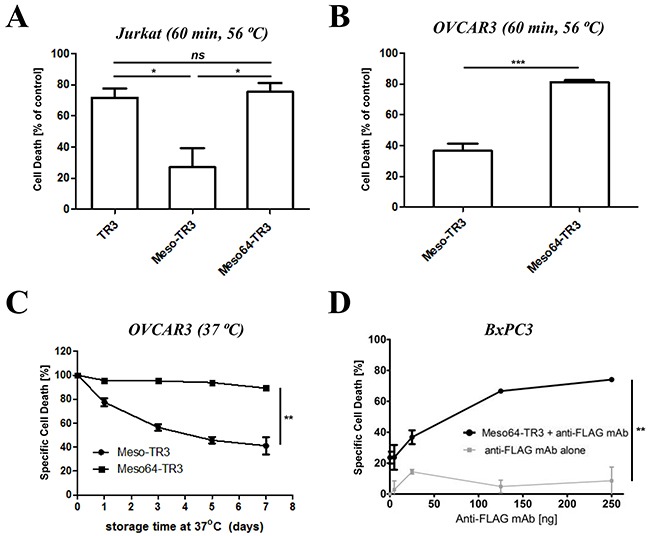
Meso64-TR3 is a temperature-stabilized monomer **A.** TR3, Meso-TR3 and Meso64-TR3 were treated at 56°C for 60 minutes and the effect on the killing capacity was evaluated on MUC16-deficient Jurkat cells (temperature influence on the TR3 effector domain). NS, not significant; *P < 0.02. **B.** TR3, Meso-TR3 and Meso64-TR3 were treated at 56°C for 60 minutes and the effect on the killing capacity was evaluated on MUC16-positive OVCAR3 cells (temperature influence on the MUC16 targeting domain effector domain). ***P < 0.0005. **C.** Meso-TR3 and Meso64-TR3 were incubated at 37°C for up to seven days and the effect on the killing capacity was evaluated on OVCAR3 cells. **P < 0.002. **D.** BxPC3 cells (MUC16-low) were treated with low-dose Meso64-TR3 (24% specific cell death) in the presence of increasing concentrations of anti-FLAG mAb to facilitate drug dimerization, which is associated with an increase in DR5 signaling and apoptosis induction. Cells treated with anti-FLAG mAb alone served as a control. **P < 0.004.

In order to complete the characterization phase of Meso64-TR3, we exposed our novel cancer drug to physiologic and elevated temperature conditions and assessed the impact of these parameters on the respective structural components of the dual-domain therapeutics (the targeting and the effector domain). Initially, we exposed TR3, Meso-TR3 and Meso64-TR3 to elevated, non-physiologic temperature conditions (60 min at 56 ºC) and studied the effects on the TR3 effector domain of the fusion proteins using MUC16-negative Jurkat cells. Under these conditions, Meso-TR3 lost more than 70% of its initial killing capacity, while Meso64-TR3 and TR3 lost less than 25% of their initial activities (Figure 4A, Jurkat). The same trend was noticed when the drugs were assessed on MUC16-expressing OVCAR3 cells. While Meso-TR3 lost nearly 64% of its baseline killing capacity, Meso64-TR3 lost only 19% (Figure 4B, OVCAR3). Both of these results further underscore the high phenotypic similarities between TR3 and Meso64-TR3, which are in contrast to the more temperature-sensitive and less active fusion protein Meso-TR3. Under less stringent temperature conditions (physiologic 37 ºC) but extended storage time (1 - 7 days), the same trend was noted. After an entire week of incubation at 37 ºC, Meso64-TR3 retained nearly all of its killing capacity on OVCAR3 cells (90%), while Meso-TR3 lost its activity quickly to only 40% of its initial potency (Figure 4C).

In our previous study, we reported on the monomeric nature of Meso-TR3, which was based on experimental evidence that following crosslinking with a mesothelin-specific monoclonal antibody, a drastic increase in bioactivity was achieved on MUC16-negative Jurkat cells [28]. Since truncated Meso64-TR3 did likely no longer contain the binding epitope for this mAb, we used an antibody directed against the FLAG epitope tag of our fusion proteins. When BxPC3 pancreatic cancer cells were treated with a sublethal dose of Meso64-TR3 (~25% cell death) in the presence of the anti-FLAG monoclonal antibody M2, we could demonstrate a dose-dependent augmentation of cell death to nearly 80% at the highest concentration of cross-linking antibody (Figure 4D). These results strongly suggest that Meso64-TR3 is indeed a monomer in solution that can be functionally enhanced by forming homodimers via antibody crosslinking.

### Targeted Meso64-TR3 selectively eliminates MUC16-positive cancer cells

**Figure 5:**
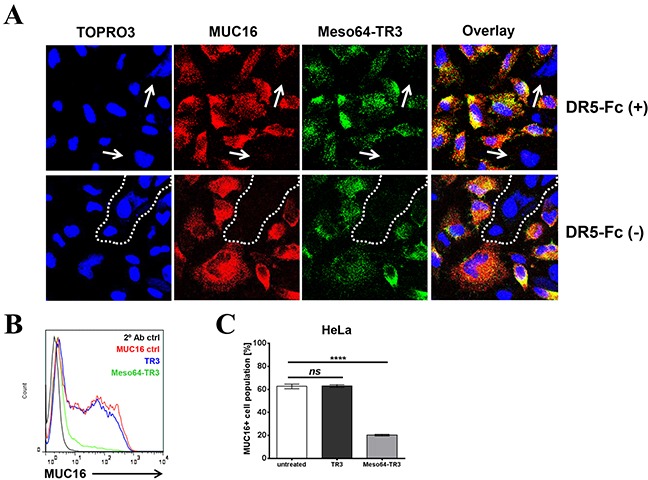
Meso64-TR3 preferentially binds to MUC16-expressing tumor cells via the high affinity mesothelin/MUC16 interaction **A.** HeLa cells were grown on 8-chamber EZ slides and incubated the following day with Meso64-TR3 complexed with and without DR5-Fc. After washing, the cells were stained with anti-MUC16 pAb (red) and anti-FLAG mAb (green), respectively. The cells were counterstained with TOPRO3 (blue, nuclei) and analyzed by confocal microscopy. The individual channels were overlaid to document co-localization of tumor marker and the targeted cancer drug (Overlay). Original magnification: 40x. **B.** HeLa cells were treated with TR3 (blue) and Meso64-TR3 (green) for 24 hours. Two days post-treatment, the cells were stained with anti-MUC16 antibody (mAb X75) and assessed for changes in the MUC16 ratio using flow cytometry. Representative histogram overlays are shown from experiments done at least twice in triplicates. **C.** Graphic representation of the data shown in (B). Error bars, mean ± SD. NS, not significant; ****P < 0.0001.

It was predicted that the enhanced killing capacity of Meso64-TR3 was mediated by a selective delivery mechanism to MUC16-positive cells, followed by induction of apoptosis through TR3/DR interaction [28]. If this was true, we anticipated that MUC16-positive cancer cells should be preferentially eliminated from a heterogeneous mix of positive and negative cells. The human cervical cancer cell line HeLa is an example of a native mix of MUC16-positive and negative cells. Confocal microscopy was employed to identify the various expression levels on different cells within the mix. After treating the cells with Meso64-TR3/DR5-Fc complexes (necessary to prevent binding directly via TR3, compare Figure 1C), we identified MUC16-negative cells that also lacked the signal for our cancer drug (Figure 5A, DR5-Fc[+], arrows).

In order to study a potential hierarchy of binding events and/or affinities of our dual-domain therapeutic (mesothelin/MUC16 *vs.* TR3/DR), we treated HeLa cells only briefly (10 min) with Meso64-TR3 in the absence of TR3-blockade (no DR5-Fc complex formation). Even under these conditions, we obtained similar staining profiles as in the presence of TR3-blockade, with areas lacking signals for both MUC16 and Meso64-TR3 (Figure 5A, DR5-Fc[−], dashed line). These data gave us first clues regarding the hierarchy of binding events and suggest that the high affinity of the mesothelin/MUC16 interaction likely dominates the TR3/DR interaction of the fusion protein and leads to a quick absorption of Meso64-TR3 by MUC16-expressing cells (see below for a more detailed analysis using an extrinsic pathway sensitizer to reveal this phenomenon).

We next used more quantitative means (flow cytometry) to document the selective killing capacity of Meso64-TR3 on MUC16-positive cancer cells. For this experiment, we treated HeLa cells, a native mix of MUC16-positive and -negative cells, with TR3 and Meso64-TR3. Several days later, the cells were analyzed for their MUC16 expression profiles. While TR3 alone was unable to alter the MUC16 ratio, similar to the untreated control cells, treatment with Meso64-TR3 decreased the number of MUC16-positive cells by nearly 70% (Figure 5B, 5C). These findings support our binding data in that the MUC16-positive cells are selectively targeted and eliminated by Meso64-TR3, a feature not shared with non-targeted TR3.

### Meso64-TR3 retains MUC16-selective killing properties in cancers refractory to TRAIL monotherapy

It is well known that not all cancers are equally responsive to TRAIL treatment. In fact, some cancers are quite resistant with regard to apoptosis induced via the extrinsic death pathway. Fortunately, an ever increasing number of sensitizing agents of the TRAIL death receptor pathway are available and have been studied extensively, including targeted SMAC mimetics [36–39]. In order to assess the potential benefit of a MUC16-targeted TR3 variant over its non-targeted counterpart on MUC16-positive cells that are refractory to TRAIL treatment, we took advantage of our recently designed, cancer-targeted small molecule SMAC mimetic SW IV-134 [40, 41]. It was predicted to be ideally suited to augment the extrinsic death pathway due to its dual activity profile involving cIAP degradation and XIAP blockade. In our current study, we explored the pathway sensitizer SW IV-134 in an effort to document the differential killing characteristics between MUC16-targeted and non-targeted forms of TR3.

**Figure 6:**
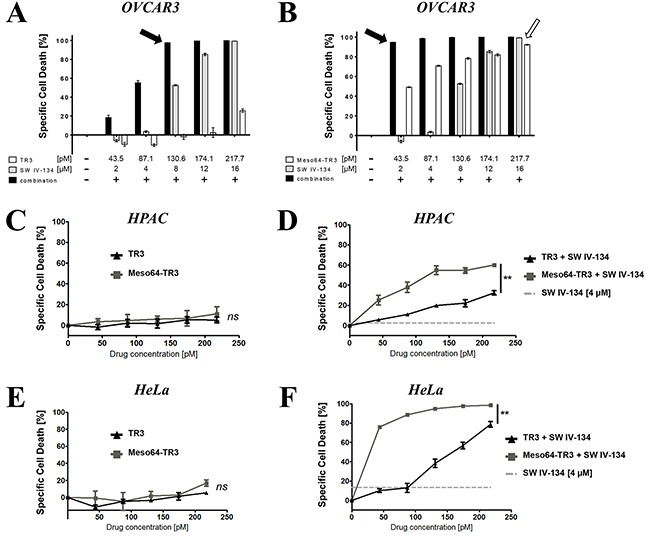
Pathway sensitization reveals the full potential of MUC16-targeted Meso64-TR3 in both TRAIL sensitive and refractory cancer cells Cell viability determinations were performed on TRAIL sensitive OVCAR3 cells **(A. and B. 100% MUC16)** and the TRAIL refractory cells HPAC **(C. and D. 50% MUC16)** and HeLa **(E. and F. 60%- 80% MUC16)**. In order to illustrate the benefit of MUC16 targeting, OVCAR3 cells were treated with TR3 **(A)**, Meso64-TR3 **(B)** and SW IV-134 **(A, B)** alone and in combination with each other at increasing concentrations of both cancer drugs. Please note that the drug concentrations required to achieve close to 100% target cell death using combination therapy (solid arrows) are much reduced for Meso64-TR3 compared to the drugs used in isolation (open arrow). The killing capacities of TR3 and Meso64-TR3 were also studied on cells that were refractory to TRAIL treatment in the absence (**C;** HPAC, NS, not significant and **E;** HeLa, NS, not significant) and in the presence of pathway sensitization using constant doses of SW IV-134 (**D;** HPAC, **P < 0.004 and **F;** HeLa, **P < 0.003).

To determine the optimal drug doses for the assessment of additive/synergistic treatment effects, OVCAR3 cells (TRAIL-sensitive, 100% MUC16+) were treated with increasing concentrations of TR3, Meso64-TR3 and SW IV-134. At picomolar concentrations, TR3 and Meso64-TR3 displayed their characteristic activity profiles with Meso64-TR3 being substantially more potent than TR3 (Figures 6A and 6B). SW IV-134 induced cell death in a dose-depending fashion consistent with previously published data and required a low micromolar concentration range [40]. When both drugs were combined additively, the cells responded to the drugs much stronger. At the lowest concentration of each agent (43.5 pM TRAIL drugs and 2 μM SW IV-134), TR3's killing capacity increased only to ~20%, while Meso64-TR3 killed more than 90% of the cells (Figures 6A and 6B). In order to achieve nearly complete tumor cell elimination following combination therapy with TR3, a 3-fold higher molar concentration of TR3 and a 4-fold higher SW IV-134 concentration were required (Figure 6A and 6B; 130.6 pM TR3 + 8 μM SW IV-134 *versus* 43.5 pM Meso64-TR3 + 2 μM SW IV-134, black arrows). Combination therapy substantially reduced the amounts of drugs needed to achieve complete tumor cell death with 5-fold less Meso64-TR3 and 8-fold-less SW IV-134 (Figure 6B, compare black and open arrow). The strong sensitizing effect of SW IV-134 during Meso64-TR3 co-treatment was suggestive of a synergistic cell death mechanism. We thus repeated this experiment by lowering both drug doses during combination treatment (Supplementary Figure S1A). Mathematical modeling using CompuSyn software [42] confirmed a synergistic rather than an additive drug effect with a combination index (CI) < 1 (range: 0.16 - 0.63) (Supplementary Figure S1B).

We next repeated the above experiment with the TRAIL-refractory cell lines HPAC (pancreatic cancer) and HeLa (cervical cancer), both comprised of a 50% - 80% mixture of MUC16-expressing cells. Even though both cell lines express varying levels of MUC16, treatment with targeted and non-targeted TR3 variants was rather inefficient and did not cause significant cell death within the indicated concentration range (Figures 6C and 6E). However, following pathway sensitization with SW IV-134 (4 μM constant dose), MUC16-targeted Meso64-TR3 outperformed its non-targeted counterpart in both instances (Figures 6D and 6F). Of note, and only following combination treatment, Meso64-TR3 selectively eliminated the MUC16-positive cells from a mixed population of HPAC pancreatic cancer cells (Supplementary Figure S2A and S2B). These data are in agreement with a selective accumulation of targeted Meso64-TR3 on biomarker-expressing cancer cells, a process that is independent of the TR3/DR interaction (Supplementary Figure S2C). Along these lines, combination treatment of pancreatic cancer cells with absent (or nearly undetectable) MUC16 expression did not result in a differential killing profile between the two TRAIL drugs (Supplementary Figure S3).

### The mesothelin/MUC16 interaction dominates the TR3/death receptor interaction of the dual-domain biologic Meso64-TR3

Functional cell viability data suggested that Meso-TR3 and Meso64-TR3 appeared to have much higher affinity to MUC16-expressing cancer cells than non-targeted TR3. However, the corresponding assays were usually performed following a 24 h drug exposure. In order to unequivocally proof this hypothesis, we designed an experiment in which OVCAR3 cells (100% MUC16-positive) were exposed for short time periods (5 - 60 min) to equimolar concentrations of the TR3 drugs (targeted or non-targeted), washed extensively to remove the non-bound biologics, followed by a 24 h exposure to the pathway sensitizer SW IV-134. This two-step design allowed us to separate initial drug binding events from pathway amplification, primarily as an experimental tool, necessary due to the limited activity profile of TR3 on OVCAR3 cells.

**Figure 7:**
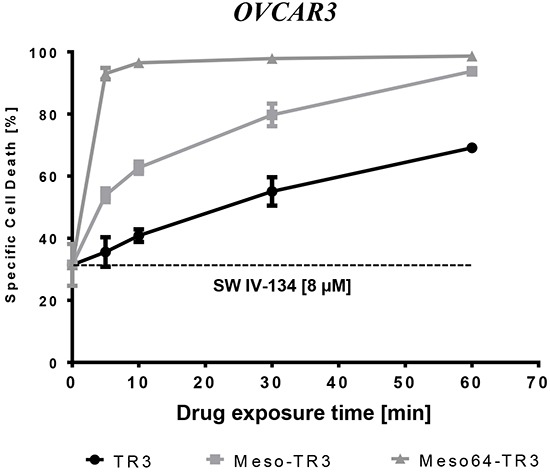
Meso64-TR3's enhanced activity profile is dominated by its much enhanced binding affinity and rapid surface tethering to MUC16-positive cancer cells OVCAR3 cells were exposed for the indicated time points to TR3, Meso-TR3 and Meso64-TR3 (218 pM each). Then, the cells were washed three times with PBS in order to remove the unbound biologics, followed by treatment of all cells with a constant dose SW IV-134 for additional 24 hours (8 μM), after which cell viability was determined. Please note that in order to achieve close to complete target cell death, Meso64-TR3 required only a five minute binding interval, not matched by TR3 and not even Meso-TR3. *P* < 0.003.

And indeed, we noticed a time-dependent increase in overall cell killing capacity for all TR3 drugs in combination with SW IV-134 (baseline activity: 31%). The most dramatic effect was seen with Meso64-TR3, approaching 95% cell death induction after only a five minute binding interval (Figure 7). Prolongation of the binding time did not increase cell death induction and suggests that Meso64-TR3 saturated the cancer cell membrane quickly within the first five minutes of drug exposure. Meso-TR3 required a 60 minute binding time to achieve maximum target cell killing (94%), followed by TR3 (can only bind via DR interaction) with a 70% killing maximum at the one hour drug exposure mark. Overall, the time-response killing curves obtained following pathway sensitization with SW IV-134 closely mimic the dose-response activity profiles for the respective TR3 drugs when used alone (compare Figure 2C) and highlight the benefits of tethering TR3-based therapeutics to the cancer cells via the high-affinity interaction between mesothelin and MUC16.

## DISCUSSION

Cancer therapy usually offers only a narrow window of opportunity when it comes to finding the most appropriate drug doses while limiting toxic side effects for the patients. In this regard, TRAIL has garnered tremendous interest as a cancer drug as it has demonstrated tumor-selective activity profiles without being toxic to the host. Along these lines, it is important to point out that the native cytokine, as well as our recently designed drug platform TR3, does not have the capacity *per se* to actively discriminate between transformed cancer cells and healthy host tissues. These non-transformed host tissues, including certain immune effector cells, have been shown to express various TRAIL receptors and act primarily as a “sink” for TRAIL-based therapeutics [43–45], but are protected from the cytokine by intracellular cFLIP expression [43]. Furthermore, other non-immune host cells, under certain inflammatory conditions, can even become susceptible to TRAIL therapy, a potential cause of undesirable side effects [46–48].

Over the years, various recombinant TRAIL variants have been engineered to enhance the stability and pharmacologic potency of the cytokine but, much to our surprise, truly cancer-targeted variants have not yet been tested in clinical trials. In order to endow the TRAIL cytokine with tumor selectivity and to minimize potential off-target toxicities, targeting moieties need to be incorporated into the therapeutics. Such downstream modifications would not only ensure accumulation of the therapeutics at the tumor site, it tethers the drugs via biomarker association to the tumor cell surface. This membrane conversion has been shown to result in far more robust death receptor signaling events than the non-targeted parental variants can ever accomplish [28, 49, 50].

In this study, we built on our previously established drug platform TR3 [25] and generated a truncation variant of Meso-TR3 [28], designated Meso64-TR3, representing a functionally improved, next generation TRAIL-based cancer therapeutic targeted to the biomarker MUC16.

Meso-TR3 is a rather bulky molecule with a molecular weight of more than 100 kDa, primarily due to its extended secondary structure (amino acid sequence), relative to the more compact structural organization of Meso64-TR3. By eliminating more than 75% of the C-terminal region of the mesothelin targeting moiety, three putative glycosylation sites were removed, which contributed significantly to the molecular weight loss relative to Meso-TR3. Also, it has been known for a while [27] and recently confirmed [35] that the amino-terminal 64 amino acids of mesothelin were sufficient to facilitate efficient binding to MUC16. Our current study was thus primarily designed as a proof-of-concept to assess if such a short peptide sequence would still facilitate binding to native MUC16, especially in the context of a fusion protein with a TR3 effector domain being nearly 6.5-fold the size of the 64 amino acid mesothelin binding moiety. We were therefore encouraged to see that Meso64-TR3's binding affinity to the MUC16 biomarker was not only retained but resulted in a much more potent cancer drug compared to its full length predecessor on MUC16-positive tumor cells. On the other hand, it turned out that Meso64-TR3 and TR3 were functionally indistinguishable on MUC16-negative cancer cells (Jurkat and other, nearly MUC16-negative cancer cells, such as BxPC3), while Meso-TR3 showed a markedly reduced activity profile on these cells, in accordance with its previously proposed prodrug feature [28]. We can only speculate about the reason(s) for Meso64-TR3 being much more potent than Meso-TR3 on MUC16-expressing cancer cells but we believe that the high sensitivity of the full length mesothelin targeting moiety to elevated (56°C) and even to physiologically more relevant temperature conditions (37°C) might be a key discriminating factor for Meso-TR3's reduced functional properties.

While in the process of characterizing this novel cancer drug candidate on a mechanistic level, we confirmed that key extrinsic pathway components were engaged following Meso64-TR3 treatment. In addition, biophysical analyses demonstrated much enhanced thermal stability of Meso64-TR3 over Meso-TR3 on MUC16-positive and -negative cancer cells, acting as a monomer in solution with a preference to eliminate the cells it binds. Along these lines, the substantially enhanced activity profile of Meso64-TR3 compared to TR3 on OVCAR3 cells is quite remarkable, given that these cells have been reported to be nearly resistant to TRAIL due to MUC16-dependent upregulation of cFLIP and a reduction in DR5 expression [51]. Thus, despite these unfavorable circumstances, we could demonstrate that membrane tethering to MUC16 with Meso64-TR3 (and Meso-TR3 to a lesser extend) could indeed overcome the therapeutic plateau observed with non-targeted TRAIL variants.

Furthermore, we shed new light on potential hierarchies with respect to epitope preferences, primarily responsible for initial drug binding events at the cancer cell membrane. Since MUC16-targeted TR3 represents a dual-domain therapeutic, at least two binding scenarios can be discriminated – 1. the mesothelin/biomarker (MUC16) interaction and 2. the TR3/death receptor interaction. In our previous study, we addressed this ambiguity by preincubating the targeted reagent (Meso-TR3) with soluble death receptor (DR5-Fc), to ensure that the binding process to MUC16 was exclusively mediated by the mesothelin targeting moiety of the fusion protein [28]. In our current study, we assessed this aspect in greater detail and included experiments in which complex formation with DR5-Fc was omitted. Confocal imaging data suggested that the strong affinity of mesothelin to MUC16, including our latest data on the 64 amino-acid truncation variant Meso64-TR3, dominated during the attachment process over the native death receptor interaction, since the staining patterns in the presence and absence of death receptor blockade turned out to be indistinguishable.

In an attempt to further confirm our imaging results and to demonstrate the advantage of using targeted versus non-targeted TR3 therapeutics for cancer therapy, we designed experiments in which the extrinsic death pathway was sensitized with an activator of the intrinsic pathway using the small molecule drug conjugate SW IV-134 [40, 41]. We could indeed show that extrinsic pathway sensitization with SW IV-134 had a much stronger effect for targeted TR3 therapy than non-targeted TR3 by shifting the therapeutic index further to lower overall drug concentrations in combination with Meso64-TR3 on various cancer cell lines exhibiting varying MUC16 expression levels. When this pathway sensitizer was incorporated in a time-dependent, “functional binding” assay, we could demonstrate that Meso64-TR3 required the shortest exposure time to achieve the highest target cell deaths, followed by Meso-TR3 and the non-targeted TR3 parent. These results highlight the positive impact of a high biomarker affinity for the development of targeted TR3-based drugs in order to ensure efficient drug accumulation on the cancer targets, thereby preventing systemic toxicities during future preclinical and clinical investigations. Along these lines, we are currently in the process of confirming the enhanced uptake properties of MUC16-targeted Meso64-TR3 by the tumors *in vivo*, in relation to its non-targeted TR3 counterpart.

And finally, another important aspect regarding the mesothelin/MUC16 interaction is its potential contribution to homotypic (tumor cell-tumor cell) and heterotypic (tumor cell-mesothelial cell) cell interactions, especially important for the peritoneal spread in ovarian cancer patients [52]. The latter type of interaction is believed to promote adherence of tumor cells to the peritoneum, resulting in metastatic spread of the primary lesion into the abdomen [27, 53, 54]. These considerations suggest that by binding to MUC16, Meso64-TR3 may also saturate and reduce or even eliminate the available binding sites on the biomarker for adhesive interactions with mesothelin-expressing normal endothelium, thereby limiting the dissemination of tumor cells in addition to augmenting TRAIL-mediated target cell death [55].

Taken together, we predict that the strong affinity of Meso64-TR3 to native MUC16, combined with its favorable thermal stability and monomeric character are key ingredients for a successful clinical application. We further anticipate that the high affinity of Meso64-TR3 to the MUC16 biomarker facilitates rapid absorption and accumulation of the systemic drug by the tumors and results in a fast clearance from the bloodstream. Once tethered to the cancer cells *in vitro* and *in vivo*, pathway sensitization is further expected to enhance treatment efficacy while systemic off-target toxicities are predicted to be kept at a minimum. Thus, systematic pharmacokinetic/ pharmacodynamic (PK/PD) studies of our targeted cancer therapeutics (biologics and small molecule conjugates) are clearly warranted to further demonstrate a treatment benefit of targeted combination therapy in preclinical animal studies in preparation for future clinical trials.

## MATERIALS AND METHODS

### Cells and reagents

All cell lines (OVCAR3, HeLa, HPAC, BxPC3, AsPC-1, HEK293T) used in the experiments were obtained from American Type Culture Collection (ATCC, Manassas, VA). Recombinant human TRAIL was purchased from Enzo Life Science (formerly BIOMOL, International, Farmingdale, NY). The sigma-2/SMAC drug conjugate SW IV-134 was synthesized as previously reported [40]. Cell viability was detected using luciferase-based readout (CellTiter-Glo, Promega, Madison, WI). Caspase activation was determined employing Caspase-Glo Assay System (Promega, Madison, WI). The pan-caspase inhibitor Z-VAD-FMK was purchased from Enzo Life Sciences (Ann Arbor, MI).

### Plasmid construction and protein production

The basic TR3 expression plasmid [25], soluble mesothelin and Meso-TR3 were generated as previously described [28]. Meso64-TR3 was generated via insertion of a 261 bp Bsi*WI*/Asp718 (compatible with Bsi*WI*) PCR fragment into the unique Bsi*WI* restriction site of the TR3 expression platform, verified by DNA sequencing. All recombinant TR3 forms, soluble mesothelin, and DR5-Fc were produced in HEK293T cells under serum-free conditions as described [25, 28]. To obtain concentrated protein stocks, the supernatants were applied to centrifugal filter devices with a 10 kDa molecular cut-off (Centricon Plus-20, Millipore, Billerica, MA). DR5-Fc was purified using Protein A columns as per the manufacturer's instructions (Pierce, Rockford, IL).

### Analysis of cell death

Cells were seeded into 96 well plates at the respective optimal densities (1×10^4^ for OVCAR3 and HeLa cells, 5×10^4^ for Jurkat cells, 2×10^4^ for BxPC3 and AsPC-1 cells, and 1.5×10^4^for HPAC cells). Treatment was initiated the following day and cell viability was determined 18 hours after treatment using CellTiter-Glo Luminescent Viability Assay according to the manufacturer's instructions (Promega Madison, WI). The treatment conditions involved the various TRAIL variants alone (TR3, Meso-TR3 and Meso64-TR3) and combinations with SW IV-134 (drug binding/combination experiments); DR5-Fc, mesothelin and Z-VAD-FMK (blocking experiments) and anti-FLAG mAb (dimer formation experiment). Data were recorded using a Multi-Detection Microplate Reader (Synergy HT, BioTek, Winooski, VT).

### Caspase activation assays

Caspase-3, -8, and -9 activities were detected in OVCAR3 cells treated with Meso64-TR3 employing a Caspase-Glo Assay System according to the manufacturer's instructions (Promega, Madison, WI). OVCAR3 cells were seeded at a density of 1×10^4^ in 96 well plates and then treated for 2, 4, 6 and 8 hours with Meso64-TR3 the following day. 100 μL of caspase reagent was added into each well, mixed for 30 seconds using a plate shaker, and incubated for 90 additional minutes at room temperature. This assay system is based on the caspase-specific substrate activation by the respective caspases. Luminescence was measured using a multi-mode microplate reader (Bio-Tek).

### Western blot analysis

OVCAR3 cells were seeded in 10 cm plates and treated for 4 hours with vehicle, TR3 or Meso64-TR3 the following day. After washing twice with PBS, the cells were lysed in RIPA lysis buffer supplemented with complete protease inhibitor cocktail (Roche, Mannheim, Germany). The lysates were centrifuged at 14,000 rpm for 15 minutes and the supernatants were collected. The samples (30 μL for each treatment condition) were run on a 4-12% Bis-Tris polyacrylamide gradient gel and transferred to a PVDF membrane using an iBlot 2 Gel Transfer Device (Life Technologies, Carlsbad, CA). The PVDF membrane was incubated at 4°C for 24 hours with a mouse anti-caspase-8 mAb (Cell Signalling Technology, Danvers, MA), followed by a 1 hours incubation at room temperature with HRP-conjugated goat anti-mouse IgG secondary antibody (Santa Cruz Biotech, Dallas, TX). Immunoreactive bands were visualized using ClarityTM Western ECL Substrate (Bio-Rad Laboratories, Hercules, CA).

### Flow cytometry

To assess the selective killing capacity of Meso64-TR3 on MUC16-positive tumor cells, HeLa and HPAC cells (1.5×10^5^/well and 3×10^5^/well, respectively) were seeded into 6-well plates for 24 hours before treatment with TR3, Meso64-TR3 or medium (control) in the absence or presence of 4 μM SW IV-134 (to amplify the TRAIL-induced extrinsic death pathway). After incubation at 37°C for 24 hours, the supernatants were replaced by fresh medium and the cells were allowed to grow to sub-confluence. Cells were washed and harvested non-enzymatically (EDTA). The cells were then incubated with anti-MUC16 mAb X75 (Abcam, Cambridge, MA), followed by staining with FITC-conjugated secondary antibody (anti-mouse IgG, Sigma-Aldrich, St. Louis, MO) and submitted to flow cytometry (FACSCalibur, BD Biosciences, San Jose, CA).

### Confocal microscopy

OVCAR3, HeLa and HPAC cells were cultured for 24 hours on millicell EZ slides (Millipore, Billerica, MA) and treated the following day. In order to prevent binding of Meso64-TR3 via TR3/death receptor interaction, Meso64-TR3 was complexed with DR5-Fc (between 5 and 30 minutes). After washing with PBS and fixation with 4% paraformaldehyde, the cells were blocked with serum-free Protein Block (Dako, Carpinteria, CA). Primary antibodies for FLAG (mouse mAb M2) and MUC16 (rabbit pAb, Sigma-Aldrich) were allowed to bind for 2 h, washed and detected with the respective secondary Abs Alexa Fluor 488 goat anti-mouse IgG (Invitrogen, Carlsbad, CA) and Alexa Fluor 555 goat anti-rabbit IgG (Invitrogen). Confocal images were taken on a Zeiss LSM 510 META Confocal Laser Scanning Microscope (Zeiss, Jena, Germany).

### Time-dependent cell death analysis

To compare the binding capacity of non-targeted and MUC16 targeted TRAIL constructs, a binding assay was performed by allowing different binding time for these three drugs. OVCAR3 cells were seeded in 96-well plates (1×10^4^/well) and cultured for 18 hours. Equimolar concentrations of TR3, Meso-TR3 and Meso64-TR3 (218 pM) were added to the wells for 5, 10, 30 and 60 minutes before the supernatants were removed and the cells were washed three times with PBS to remove traces of unbound drugs from the wells. Due to the limited activity of TR3 on OVCAR3 cells, the pathway sensitizer SW IV-134 (8 μM) was subsequently added to all wells. Cell death was analyzed using CellTiter-Glo Luminescent Viability Assay as described above.

### Animals

Six to eight week old female Severe Combined Immunodeficiency mice (SCID; Harlan, IN) were used as hosts for tumor xenografts. Human OVCAR3 tumor pieces (2 × 2 mm^2^) were implanted into the flanks and allowed to engraft until reaching a volume of 100 mm^3^ prior to grouping and drug treatment (655 pmoles/mouse/day for 13 days). Procedures involving mice were approved by the Washington University Animal Studies Committee and conducted in accordance with the guidelines for the care and use of laboratory research animals established by the NIH.

### Statistical analyses

Treatment efficiency of *in vitro* killing assays and *in vivo* tumor growth rates are presented as means ± SEM. Statistical significance for all analyses is defined as *P < 0.05* and was calculated employing analysis of variance (one-way ANOVA, Tukey's Multiple Comparison Test) and the Student's t-test (unpaired) as indicated using GraphPad Prism (V 4.02) software.

## SUPPLEMENTARY FIGURES


