# Supplementary material for: Mesothelin's minimal MUC16 binding moiety converts TR3 into a potent cancer therapeutic via hierarchical binding events at the plasma membrane
Source: Oncotarget. 2016 Apr 22;7(21):31534–49. doi: 10.18632/oncotarget.8925 (PMC5058776; doi:10.18632/oncotarget.8925)
Supplement: Supplementary file 1 [file oncotarget-07-31534-s001.pdf]

SUPPLEMENTARY FIGURES

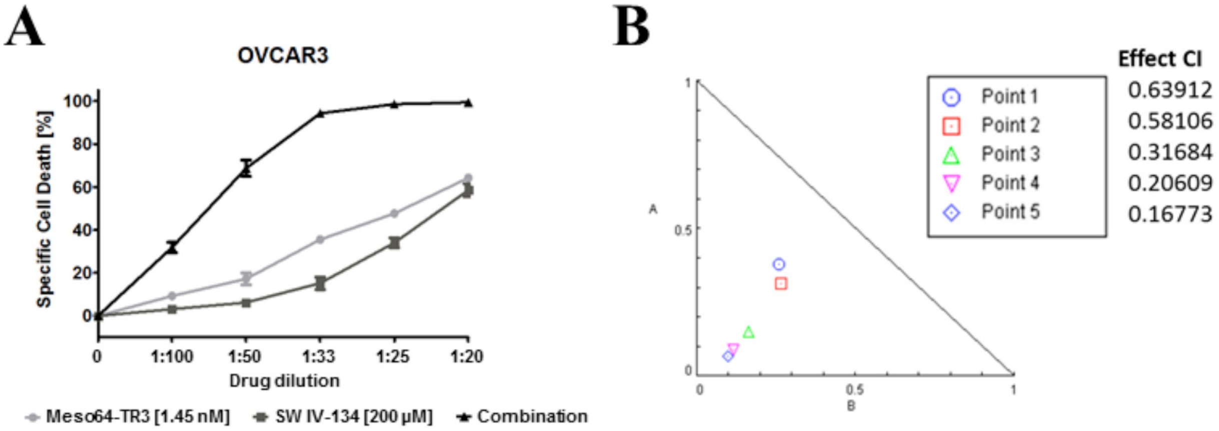

Supplementary Figure S1: Meso64-TR3 and SW IV-134 display synergistic treatment benefit on MUC16-positive cancer cells. A. A viability assay on OCVAR3 ovarian cancer cells was employed to assess the synergistic treatment potential of Meso64-TR3 and SW IV-134 using sublethal concentrations of either cancer drug alone and in combination with each other. **B.** The treatment effect was evaluated using CompuSyn software (42), with a combination index (CI) < 1 representing synergistic drug activities, identified for all five drug concentrations.

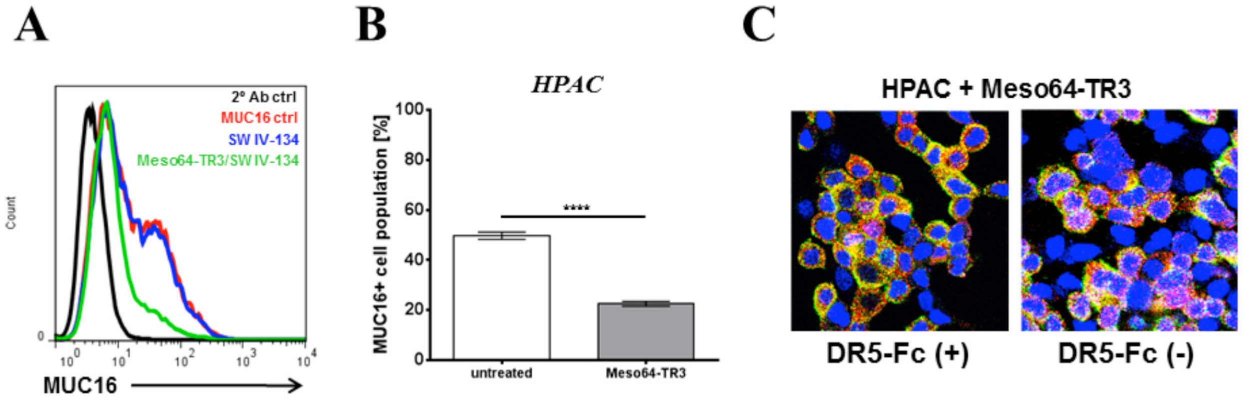

Supplementary Figure S2: Meso64-TR3 preferentially kills MUC16-expressing pancreatic cancer cells following pathway sensitization with SW IV-134. A. HPAC cells were either untreated (black and red), treated with SW IV-134 alone (blue) or with a combination of Meso64-TR3 and SW IV-134 (green) for 24 hours. Two days post-treatment, the cells were stained with MUC16 antibody (mAb X75) and assessed for changes in the MUC16 ratio using flow cytometry. Representative histogram plots are shown from experiments done at least twice in triplicates. **B.** Graphic representation of the data shown in (A). Error bars, mean ± SD. ****$P < 0.0001$. **C.** HPAC cells were grown on 8-chamber EZ slides and incubated the following day with Meso64-TR3 complexed with and without DR5-Fc (+ and -). After washing, the cells were stained with a mixture of anti-MUC16 pAb (red) and anti-FLAG mAb (green), respectively. The cells were counterstained with TOPRO3 (blue, nuclei) and analyzed by confocal microscopy. The individual channels were overlaid to document co-localization of tumor marker and the targeted cancer drug (Overlay). Original magnification: 40x.

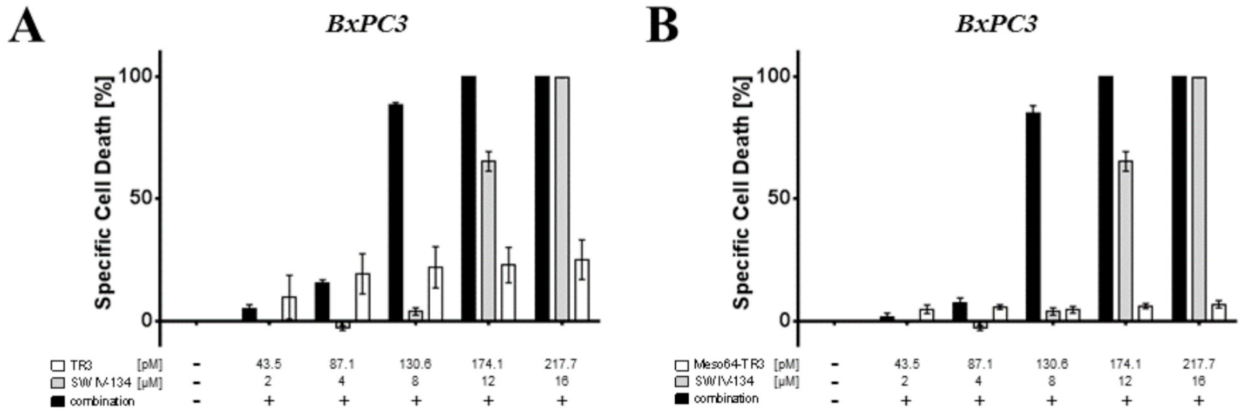

Supplementary Figure S3: Meso64-TR3 is phenotypically indistinguishable from non-targeted TR3 on pancreatic cancer cells exhibiting low MUC16 expression. Cell death assays on MUC16-low expressing BxPC3 cells to assess the killing capacity of TR3 **A.** and Meso64-TR3 **B.** alone and in combination with the pathway sensitizer SW IV-134. Please note that the activity profiles for both cancer drugs are virtually identical and reflect the low frequency and abundance of MUC16 expression on BxPC3 cells, similar to what we have demonstrated in Jurkat cells (MUC16-deficient).
